# ICTV Virus Taxonomy Profile: Endornaviridae

**DOI:** 10.1099/jgv.0.001277

**Published:** 2019-06-11

**Authors:** Rodrigo A. Valverde, Mahmoud E. Khalifa, Ryo Okada, Toshiyuki Fukuhara, Sead Sabanadzovic, ICTV Report Consortium

**Affiliations:** 1 Department of Plant Pathology and Crop Physiology, Louisiana State University Agricultural Center, Baton Rouge, LA 70803, USA; 2 Department of Botany and Microbiology, Faculty of Science, Damietta University, New Damietta City, Damietta 34517, Egypt; 3 Horticultural Research Institute, Ibaraki Agricultural Center, 165-1 Ago, Kasama 319-0292, Japan; 4 Department of Applied Biological Sciences and Institute of Global Innovation Research, Tokyo University of Agriculture and Technology, 3-5-8 Saiwaicho, Fuchu, Tokyo 183-8509, Japan; 5 Department of Biochemistry, Molecular Biology, Entomology and Plant Pathology, Mississippi State University, Mississippi State, MS 39762, USA

**Keywords:** *Endornaviridae*, ICTV Report, taxonomy

## Abstract

The family *Endornaviridae* includes viruses with linear, single-stranded, positive-sense RNA genomes that range from 9.7 to 17.6 kb and have been reported infecting plants, fungi and oomycetes. The family consists of two genera, *Alphaendornavirus* and *Betaendornavirus,* into which viruses are classified based on their genome size, host and presence of unique domains. *Alphaendornavirus* includes species whose members infect plants, fungi and oomycetes, while the genus *Betaendornavirus* includes species whose members infect ascomycete fungi. This is a summary of the ICTV Report on the family *Endornaviridae,* which is available at www.ictv.global/report/endornaviridae.

**Table 1.** Characteristics of members of the family *Endornaviridae*

Typical member:	Oryza sativa endornavirus Nipponbare (D32136), species *Oryza sativa alphaendornavirus,* genus *Alphaendornavirus*
Virion	No true virions are associated with members of this family
Genome	Monocistronic single-stranded positive-sense RNA of 9.7–17.6 kb
Replication	Cytoplasmic
Translation	From monocistronic positive-sense RNA
Host range	Host-specific; plants, fungi and oomycetes
Taxonomy	Realm *Riboviria*. Two genera including more than 20 species

## Virion

No true virions are associated with members of this family since their genomes lack a coat protein gene (Table 1). However, the RNA genome has been associated with pleomorphic cytoplasmic membrane vesicles for Vicia faba endornavirus [1].

## Genome

**Fig. 1.**
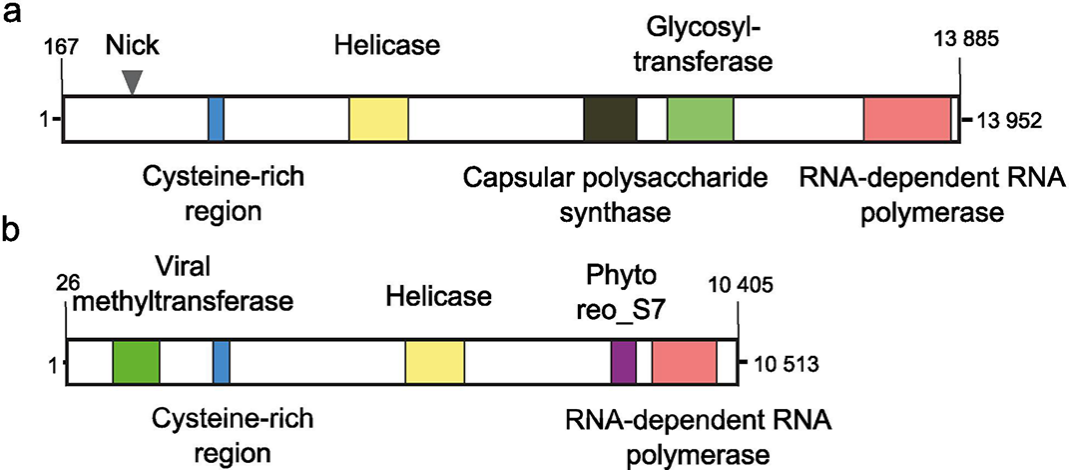
Genome organization of Oryza sativa endornavirus Nipponbare (a) and Sclerotinia sclerotiorum endornavirus 1 (b), representative members of the genera *Alphaendornavirus* and *Betaendornaviru*s, respectively. Numbers indicate genome nucleotide positions.

The genome of endornaviruses consists of a linear, single-stranded RNA containing a single ORF encoding a polyprotein that ranges from 3217 to 5825 aa [2,4]. The terminal sequence of the 3′-end of most endornavirus RNAs consists of poly(C). A conserved RNA-dependent RNA polymerase domain located in the C-terminal region of the polyprotein is a feature common to all endornaviruses. Other domains found in some, but not all, endornaviruses include viral helicase superfamily 1, methyl transferase, glycosyl transferase, cysteine-rich region, phytoreo_S7 domain and capsular polysaccharide synthase (Fig. 1) [2,5]. It is likely that the ORF also encodes one or more proteinases to cleave the encoded polyprotein into functional proteins [2].

## Replication

**Fig. 2.**
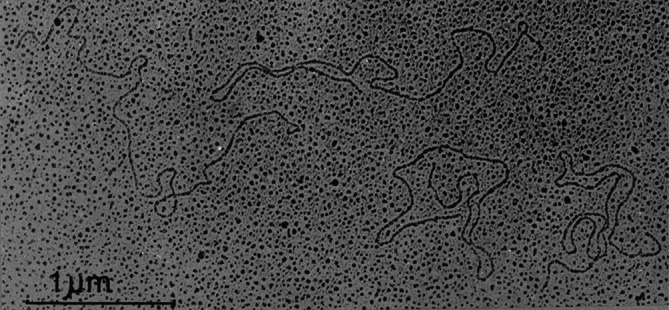
Transmission electron micrograph of dsRNA molecules of Oryza sativa endornavirus isolate Nipponbare. Figure reproduced with permission from [7].

Most endornaviruses have been characterized using the viral replicative forms (dsRNAs), which are relatively stable, present in relatively high quantities in the host tissue, and easily isolated (Fig. 2). RNA-dependent RNA polymerase activity associated with Oryza sativa endornavirus has been detected in the crude microsomal fraction of rice cultured cells and with cytoplasmic vesicles containing Vicia faba endornavirus RNA [1,6].

## Biology

Endornaviruses have been reported in several economically important crops and their wild relatives, in plant pathogenic fungi, and in species of the oomycete *Phytophthora* [2]. Plant endornaviruses are transmitted only through the gametes [2]. Because of their persistent lifestyle and lack of effect on the host phenotype (with some exceptions), limited information is available about the biology of these viruses.

## Taxonomy

### Alphaendornavirus

Alphaendornaviruses infect plants, fungi and the oomycete *Phytophthora* sp. [2]. They are characterized by longer genomes (>11.9 kb) than members of the genus *Betaendornavirus*. The majority of alphaendornaviruses contain a glucosyltransferase domain and a site-specific nick near the 5′-end of the coding strand of the replicative form.

### Betaendornavirus

Betaendornaviruses infect ascomycete fungi [8]. They differ from alphaendornaviruses in having shorter genomes (<10.7 kb), lacking a glucosyltransferase domain or 5′-terminal site-specific nick, but having a methyltransferase domain.

## Resources

Full ICTV Report on the family *Endornaviridae*: www.ictv.global/report/endornaviridae.

## References

[1] Lefebvre A, Scalla R, Pfeiffer P (1990). The double-stranded RNA associated with the '447' cytoplasmic male sterility in *Vicia faba* is packaged together with its replicase in cytoplasmic membranous vesicles. Plant Mol Biol.

[2] Fukuhara T, Gibbs MJ, King AMQ, Adams MJ, Carstens EB, Lefkowitz EJ (2012). Virus Taxonomy: Classification and Nomenclature of Viruses: Ninth Report of The International Committee on Taxonomy of Viruses.

[3] Stielow B, Klenk H-P, Menzel W (2011). Complete genome sequence of the first endornavirus from the ascocarp of the ectomycorrhizal fungus *Tuber aestivum* Vittad. Arch Virol.

[4] Gibbs MJ, Koga R, Moriyama H, Pfeiffer P, Fukuhara T (2000). Phylogenetic analysis of some large double-stranded RNA replicons from plants suggests they evolved from a defective single-stranded RNA virus. J Gen Virol.

[5] Sabanadzovic S, Wintermantel WM, Valverde RA, McCreight JD, Aboughanem-Sabanadzovic N (2016). Cucumis melo endornavirus: genome organization, host range and co-divergence with the host. Virus Res.

[6] Horiuchi H, Udagawa T, Koga R, Moriyama H, Fukuhara T (2001). RNA-dependent RNA polymerase activity associated with endogenous double-stranded RNA in rice. Plant Cell Physiol.

[7] Fukuhara T, Moriyama H, Pak JY, Hyakutake H, Nitta T (1993). Enigmatic double-stranded RNA in Japonica rice. Plant Mol Biol.

[8] Khalifa ME, Pearson MN (2014). Molecular characterisation of an endornavirus infecting the phytopathogen *Sclerotinia sclerotiorum*. Virus Res.

